# Clinical Relevance of +936 C>T *VEGFA* and c.233C>T *bFGF* Polymorphisms in Chronic Lymphocytic Leukemia

**DOI:** 10.3390/genes11060686

**Published:** 2020-06-23

**Authors:** Sandra Ballester, Begoña Pineda, Patricia Rodrigues, Eduardo Tormo, María José Terol, Pilar Eroles

**Affiliations:** 1 Biomedical Research Institute INCLIVA, 46010 Valencia, Spain; sandra.ballester@uv.es (S.B.); begona.pineda@uv.es (B.P.); patriciaels@gmail.com (P.R.); eduardo.tormo@uv.es (E.T.); maria.jose.terol@uv.es (M.J.T.); 2 Departament of Phisiology, University of Valencia, 46010 Valencia, Spain; 3 Centro de Investigacion Biomedica en Red Cancer (CIBERONC), 28029 Madrid, Spain; 4 Hematology Department, Hospital Clínico Universitario de Valencia, 46010 Valencia, Spain

**Keywords:** polymorphisms, chronic lymphocytic leukemia risk, vascular endothelial growth factor, fibroblast growth factor

## Abstract

Angiogenesis process contributes to the pathogenesis of B-cell chronic lymphocytic leukemia (B-CLL) being the levels of VEGFA and bFGF higher in patients than in healthy controls. Our aim was to evaluate the implication of angiogenesis factors genetic variants in the predisposition to B-CLL and their association with clinical factors and survival. We performed a population-based case-control study in 224 Spanish B-CLL patients and 476 healthy randomly selected controls to evaluate susceptibility to developing B-CLL. Six polymorphisms were evaluated: rs1109324, rs1547651, rs3025039 (+936 C>T), rs833052 of the *VEGFA* gene, rs1449683 (c.233C>T) of the *bFGF* gene and (−710 C>T) of the *VEGFR1* gene. The association between clinical parameters and patient outcome was analyzed. Carriers of the CT/TT variants of rs3025039 showed a significant protective effect against developing B-CLL. The CT/TT variants of rs1449683 show a tendency towards the development of the disease and the same variants associated significantly with higher genetic risk and with reduced disease free survival. Moreover, the association persisted in the early-stage disease subgroup. Our study provides evidence of the protective effect of the T/- rs3025039 *VEGFA* variant against B-CLL development and the association of CT/TT variants of the rs1449683 *bFGF* gene with genetic risk and an adverse survival.

## 1. Introduction

B-cell chronic lymphocytic leukemia (B-CLL) is characterized by the accumulation of mature monoclonal B cells in the blood, secondary lymphoid tissue and marrow [1,2]. B-CLL is a heterogeneous disease with a highly variable clinical outcome. The incidence varies between geographical areas being the most common leukemia in the western world. An incidence of about 19,000 new cases in the US was estimated in 2016 [3].

Angiogenesis is an essential process in the development and growth of malignant tumors, and several studies indicate that it may also be involved in the pathogenesis of B-CLL. This is a multistep process requiring integrated actions of a number of angiogenesis growth factors [4], among which the most potent inducers are the fibroblast growth factor (bFGF) and the vascular endothelial growth factor (VEGF) family [5,6,7]. The different VEGFs (VEGFA, VEGFB, VEGFC, VEGFD and VEGFE) and its receptors (VEGFR1, VEGFR2 and VEGFR3) and bFGF play key roles in neovascularization and maintenance of the adult vasculature [8]. B-CLL cells are able to produce both pro-angiogenesis and anti-angiogenic factors [9]. Several authors have shown that VEGF and bFGF serum levels are higher in B-CLL patients than in normal subjects [9,10,11,12], and that all three VEGF receptors are expressed in B-CLL cells [13]. Recent studies have identified regulatory elements differently activated in B-CLL and normal cells, including the hypoxia inducible factor (HIF), a transcription factor that activates angiogenesis factor expression under hypoxia conditions. Furthermore, it has been shown that transcription factor binding sites could be frequently changed by single nucleotide polymorphism (SNP) modifications [14].

Multiple SNPs have been identified within the *VEGFA* gene. Among them, the +936 C>T polymorphism (rs3025039) has been studied extensively in many cancers [15,16,17,18,19,20,21] showing association with cancer risk. However, current results for B-CLL are limited and show either no significant association [22] or just one trend [23] with B-CLL predisposition.

The FGF family play key roles in neovascularization, wound healing and cancer [10]. Among the SNPs identified in *bFGF*, rs1449683 (c.233C>T), located in exon 1, has been found to be associated with the gene expression both transcriptionally and translationally. In particular, the C/C genotype of the rs1449683 *bFGF* polymorphisms has been related with both elevated mRNA and protein levels [24].

The association of polymorphisms genotypes with cells functions has been proved in patients with B-CLL [25]. We hypothesized that SNPs in angiogenesis pathway genes could modify B-CLL risk. We performed a population-based case-control study to investigate the possible modifying effect of four polymorphisms in *VEGFA*, one in *VEGFR1* and another in *bFGF*. Subsequently, we also examined the relationship between *VEGFA, VEGFR1* and *bFGF* polymorphisms variants and prognostic factors, the clinical parameters of the disease and the relationship with survival in B-CLL patients.

## 2. Materials and Methods

### 2.1. Study Participants

Two hundred and twenty-four B-CLL patients with different clinical/hematological features from the University Clinic Hospital of Valencia in Spain were included in our analysis. All the patients in the study had typical B-CLL (mature lymphocytes expressing CD5, CD19, CD23 and clonally restricted surface immunoglobulin) and were selected on the basis of the white blood cell (WBC) count, mostly >20 × 10^9^/L. B-CLL diagnosis was based upon standard morphological and immunophenotypic criteria. For retrospective analysis, and according to the study inclusion criteria, samples from patients between 1986 and 2012 (with an average of 67 ± 11 years) were selected and processed. Patient characteristics are detailed in Table 1. Genetic abnormalities were detected by conventional cytogenetic and FISH analysis including probes for detection of deletions of 13q14 (D13S25 and D13S319), trisomy 12, 11q22.3 (ATM) and 17P13 (TP53) [26]. The mutational status of the IgVH gene was analyzed according to European Research Initiative on B-CLL recommendations [27]. Zap-70 and CD38 expression was analyzed by flow cytometry. Clinical pathological parameters were obtained from hospital clinical records. Mean follow-up of patients was 6.7 ± 4.7 years.

We also conducted a control group study, analyzing a total of 476 healthy cases with a mean age of 44 ± 13 years. The control population without previous or concurrent malignant disease was collected for the same time periods as the cases. Samples were from the blood donor bank of the same hospital. Peripheral blood mononuclear cells from whole blood were isolated using density gradient centrifugation (Lymphoprep™; Thermo Fisher Scientific, Waltham, MA, USA) before cryopreservation in liquid nitrogen.

All study participants gave their informed consent and the study was approved by the institution’s Research Ethics Committee.

### 2.2. Selection of SNPs for Study

Candidate SNPs were selected using a bioinformatic approach (dbSNP (NCBI), SNPselector and SYSNPs) and according to the bibliography data. SNPs associated with altered *VEGFA* and *bFGF* expression in B-CLL [28,29,30] and prognostic significance in other malignancies [19,20,31] were chosen. A total of six SNPs were selected (Table 2): four 3′UTR polymorphisms, one polymorphism in the promoter region of the *VEGF* gene and one polymorphism at the cds-synon region of the *bFGF* gene.

### 2.3. DNA Extraction and Genotyping

Briefly, DNA extraction was carried out from 10 mL of venous blood, using EDTA-containing tubes. DNA isolation kit and QIAcube protocol by QIAGEN^®^ were used. A final elution volume of 100 μL was established. DNA quantity was measured by absorbance at 260 nm using a NanoDrop spectrophotometer, and DNA purity was evaluated by measurement of 260/280 absorbance ratios. Each DNA sample was stored at −20 °C until analysis.

Genotyping analyses of the SNPs were performed by real-time PCR, using the TaqMan^®^ SNP Genotyping Assays (Thermo Fisher Scientific, Waltham, MA, USA). Assays were performed according to the manufacturer’s instructions. Briefly, each sample reaction was composed of 2.5 μL of TaqMan Genotyping Master Mix (Thermo Fisher Scientific, Waltham, MA, USA), 0.12 μL of TaqMan probe assay 40× and 15 ng of DNA. All assays were carried out in 384-wells plates and we used a triplicate for each sample. Thermal cycler conditions were 50 °C for 2 min, 95 °C for 10 min and a third stage consisting of 45 cycles of 95 °C for 15 s and 60 °C for 1 min. In order to validate this methodology, 10% of the samples were analyzed twice in independent experiments and there was a 100% concordance between both data. Thermal cycling and detection were performed on the ABI Prism 7900 and the results were analyzed using the allelic discrimination assay program of Sequence Detection Software version 2.4 (Applied Biosystems, part of Thermo Fisher Scientific, Waltham, MA, USA).

### 2.4. Statistical Analysis

Hardy–Weinberg equilibrium (HWE), genotype distributions, haplotype frequencies, association tests and linkage disequilibrium (LD) analysis were estimated using the SNPStats web statistical methodologies tool [32]. For each polymorphism, HWE was tested by comparing the observed to expected genotype frequencies in controls using a χ^2^ test. A 5% level of significance was used in the analysis. Chi-squared analyses were used to determine the differences in distribution of the *VEGFA* and *bFGF* genotype between cases and controls. Odds ratio (OR) values and the 95% confidence interval (CI) for the relative risk were calculated as a measure of the association between the different genotypes and B-CLL risk. Akaike’s information criterion (AIC) and Bayesian information criterion (BIC) were calculated to select the best inheritance model for each specific polymorphism, the preferred model being the one with the lowest AIC and BIC values.

The association between the SNPs and clinical and genetic variables of B-CLL patients (age, gender, hematologic values at diagnosis, cytogenetic abnormalities, disease status, Rai classification and Binet stage, CD38 and Zap-70 expression, lymphadenopathies, morphology and IgVH mutational status) was calculated using the χ^2^ test and by testing the Spearman’s rank correlation. Statistical data analyses were performed using the SPSS (IBM SPSS Statistics 22.0 software). All *p*-values were considered significant at *p*-value < 0.05.

Overall survival (OS) was defined as time from diagnosis to death due to disease or end of follow-up (censored observation). Disease-free survival (DFS) was defined as the interval between the diagnostic date and either the occurrence of an event (death, recurrence or progression of disease) or the time of the patient’s last clinical evaluation. Probabilities of OS and DFS were calculated according to the Kaplan–Meier method, while significant differences between survival curves were evaluated with Mantel’s log rank test. The analysis included the patients with available clinical follow-up (median 80.48 ± 56.42 months).

## 3. Results

### 3.1. Association between VEGFA, VEGFR1 and bFGF Genotypes and B-CLL Risk

Genotype frequencies of the SNPs evaluated in the *VEGFA*, *VEGFR1* and *bFGF* genes were compared between 224 B-CLL cases and 476 healthy donors. The SNPs were successfully genotyped in more than 95% of the study samples and frequencies were adjusted for age and sex. Data are summarized in Table 3.

Genotypic distribution of the candidate SNPs within the control group was under the Hardy–Weinberg equilibrium (HWE) with a value of *p* > 0.05. The polymorphic positions were named according to their reference sequence (VEGFA NM_001171630.1, VEGFR1 NM_002019.4 and bFGF NM_002006.4)

Regarding the +936 C>T rs3025039 polymorphism (*VEGFA* gene), genotype frequencies differed between B-CLL patients and controls. Carriers of the +936 C/T heterozygote and the +936 T/T homozygote variants were observed at lower frequency in the patients (16.1%) compared with the respective control group (24.6%). The +936 C/T+T/T genotypes associated significantly with a protective effect against developing B-CLL (*p*-value = 0.0075; OR = 0.61, 95% CI: 0.42–0.89).

We established haplotypes for the four polymorphisms in the *VEGF* gene in the 670 individuals with available information for all variants to assess the prognostic importance in the risk of developing B-CLL. The estimation of the frequency for each haplotype was performed with HaploView 4.2 software (Daly Lab, Cambridge, MA, USA) with the online tool SNPstats (https://www.snpstats.net/start.htm). Distribution of haplotypes was compared in case and control groups with chi-squared tests in HaploView. Permutation tests were used to correct multiple testing errors with 100,000 simulations. Adjusted ORs and 95% CIs were computed for each haplotype and compared to the most common haplotype with SNPStats. Haplotypes with frequencies greater than 1% were considered (5 from the 16 possible). The risk associated with the various haplotypes was compared with each other, taking as reference the most frequent. Two haplotypes accumulated a frequency of 74% being the CGAC haplotype the most frequent and the one that was taken as a reference. Compared with the major haplotype CGAC, the TGAC, CTTC and CGAA haplotypes appeared to be significantly reduced in patients with B-CLL compared to control individuals (OR: 0.49, 95% CI: 0.31–0.79, *p* = 0.0036; OR: 0.17, 95% CI: 0.08–0.36, *p* < 0.0001 and OR: 0.21, 95% CI: 0.10–0.41, *p* < 0.0001, respectively). The three haplotypes show significant association with a reduced risk of developing B-CLL (Table 4).

Moreover, a greater frequency of the T allele (rs1449683) of the *bFGF* gene was observed in our cohort of patients compared with controls, without reaching statistical significance (*p*-value = 0.0630; OR = 1.62, 95% CI: 0.98–2.66).

### 3.2. Correlation of VEGFA, VEGFR1 and bFGF Genotypes with Clinical/Pathological Factors of B-CLL Patients

To investigate the possible biological influence of *VEGFA*, *VEGFR1* and *bFGF* SNPs in B-CLL malignancy, we performed an analysis of SNP genotypes versus the different molecular and clinical B-CLL markers characteristic of patients according to cytogenetic abnormalities (very low risk (del13q), low risk (normal karyotype, trisomy 12), intermediate risk (del11q) and high risk (del17p)), disease status (Rai classification and Binet stage), CD38 and Zap-70 expression, IgVH mutational status, gender, age at diagnosis, lymphadenopathy, morphology, beta2 microglobulin serum level, LDH level, Hb level, platelet and WBC count at diagnosis (Table 5).

The rs1449683 *bFGF* polymorphisms and the rs1109324 *VEGFA* polymorphism showed association with cytogenetic risk (*p*-value = 0.025; OR = 4.46 (95% CI: 1.31–15.23) and *p*-value = 0.021; OR = 0.16 (95% CI: 0.02–1.21), respectively).

In addition, the rs3025039 *VEGFA* polymorphism showed association with low hemoglobin in the blood at the time of diagnosis in our patient group (*p*-value = 0.019; OR = 3.91 (95% CI: 0.51–7.30). No more significant interactions were observed between the covariates studied and this polymorphism.

The rs1547651 *VEGFA* polymorphism showed association with several factors including Rai and Binet stages (*p*-value = 0.0058; OR = 0.38 (95% CI: 0.18–0.79) and *p*-value = 0.0059 OR = 0.13 (95% CI: 0.02–0.97), respectively), the presence of lymphadenopathies (*p*-value = 0.021; OR = 0.44 (95% CI: 0.21–0.92)) and hemoglobin levels (*p*-value < 0.0004; OR = 4.90 (95% CI: 2.24–7.57)) at diagnosis. Furthermore, rs1109324 associated with cytogenetic risk (*p*-value = 0.020; OR = 0.16 (95% CI: 0.02–1.22)) and specifically with the presence of del13q (*p*-value = 0.039).

### 3.3. Correlation of VEGFA, VEGFR1 and bFGF SNPs with Prognosis of B-CLL Patients

**Figure 1:**
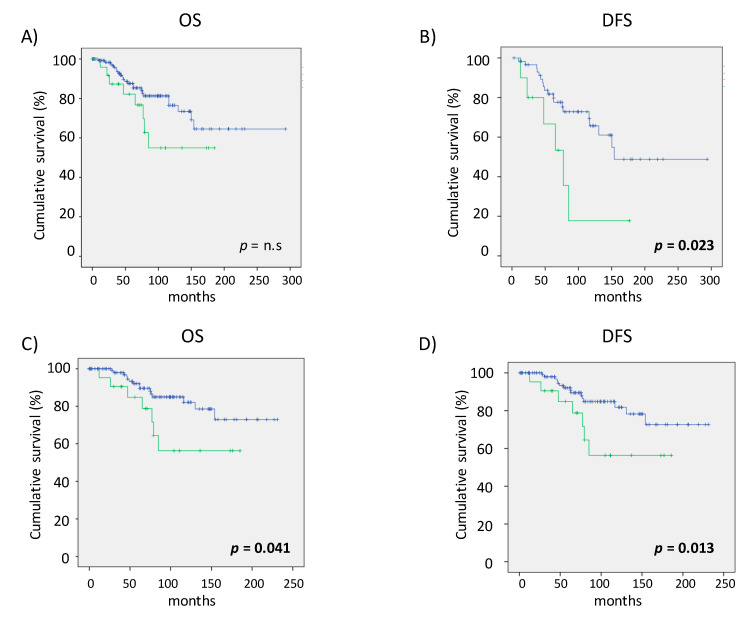
Association of the rs1449683 *bFGF* genotype with overall survival (OS) and disease free survival (DFS) in B-CLL patient groups. Kaplan–Meier curves according to the *bFGF* (T/-) genotype (green) versus other genotypes (C/C) (blue). (**A**) Overall survival (OS) and (**B**) disease free survival (DFS) in the global group of B-CLL patients (Log Rank Mantel-Cox *p*-value = n.s and *p*-value = 0.023, respectively), (**C**) OS and (**D**) DFS in patients with Binet A stage; *p*-value = 0.041; *p*-value = 0.013.

The association between individual SNP and patient outcome was assessed in patients with available information (*n* = 135). Only the rs1449683 *bFGF* gene variants showed significant association with survival. Patients carrying the T (C/T or T/T; *n* = 21) allele had worse survival than C/C carriers (*n* = 114) with a significant difference in DFS (*p*-value = 0.023). Furthermore, the survival analysis presents significance for rs1449683 polymorphisms in patients with early stage disease (initial clinical stage, Binet A classification), where the carriers of one T show the worst survival: OS (*p*-value = 0.041) and DFS (*p*-value = 0.013). Kaplan–Meier curves of rs1449683 *bFGF* genotype, OS and DFS are shown in Figure 1. Log-Rank *p*-values are displayed.

## 4. Discussion

Angiogenesis factors are believed to be important in cancer through various mechanisms, representing a key step in the emergence, progress and prognosis of different tumors [33,34]. *VEGFs* and *VEGFR* polymorphisms have been related with susceptibility for prostate, colorectal and breast cancer development with contradictory results [35,36]. Concerting B cell lymphomas some interesting results have associated particular polymorphisms in *VEGFs* or *VEGFRs* genes with the survival of patients. Indeed, Kim and co-workers found that rs1870377T>A of the *VEGFR2* gene significantly associated with OS and progression-free survival in patients with diffuse large B cell lymphoma [37]. Others studies have showed a strong correlation between complete cytogenetic response or treatment failure and the genotype of *VEGFR2* polymorphisms in chronic myeloid leukemia. In acute myeloid leukemia the genotype +936 CC/CT (rs3025039) of *VEGFA* have shown correlation with favorable leukemia survival [17].

Genetic variants of angiogenesis factors can influence the expression of the corresponding proteins, and thus modify susceptibility to and severity of cancer. Indeed, some authors suggest an association between polymorphisms and predisposition to B-CLL development [38,39] and formation of specific chromosomal aberrations [40]. Other researchers have described some polymorphisms variants linked with the prediction of clinical outcome [41].

In the present study, four polymorphisms of the *VEGFA* gene (rs3025039, rs1109324, rs1547651 and rs833052), the polymorphism located in the promoter of the *VEGFR1* gene (−710 C>T), and the polymorphism in the *bFGF* gene (c.233C>T) were analyzed in order to determine whether the presence of allelic variants are associated with susceptibility to B-CLL and to patient evolution.

Our results showed that the +936T/- rs3025039 (*VEGFA*) carriers have a significant reduced predisposition to B-CLL development. These results are consistent with previous studies that report a protective association between the +936 T/- rs3025039 carriers and other cancers [19,42,43]. We also observed an increased frequency of the T allele in the rs1449683 *bFGF* SNP in patients compared to the control group. Our data suggest that while the presence of the T allele of rs1449683 *bFGF* polymorphism may contribute to susceptibility to B-CLL, the presence of the T allele of rs3025039 *VEGFA* polymorphisms was associated with a protective effect against the disease. Additionally, the TGAC, CTTC and CGAA haplotypes of the four *VEGFA* SNPs analyzed were associated with protection against disease development. Altogether, these findings suggest that *VEGFA* SNPs are B-CLL susceptibility markers, as has been described in other cancers [17,19,44,45].

Although the rs1449683 *bFGF* SNP did not reach statistical significance as a B-CLL predisposition factor, this SNP associated significantly with high genetic risk (a very relevant marker of B-CLL disease progression) in patients. However, no correlation was found between the rs3025039 *VEGFA* SNP and the main clinical features of B-CLL. The study provides the first evidence that +936 C>T *VEGFA* and c.233C>T *bFGF* polymorphisms may influence the risk of B-CLL, since the results show that both are closely related to protective phenotype, and increased overall and disease free survival prediction, which could be applied as novel biomarkers to identify individuals at high risk of the disease. 

In addition, and more importantly, the B-CLL patient carrying the T/- (CT and TT) variants of the rs1449683 *bFGF* polymorphisms showed a significant reduction in survival in our study. Interestingly, the same genetic variants showed higher expression in patients than in healthy controls when predisposition was analyzed. Nonetheless, it did not reach statistical significance; this may be due to the low prevalence of the variant.

The variants C/T and T/T of the rs1449683 *bFGF* polymorphisms have been associated with lower mRNA and protein bFGF expression than the CC homozygote variant [24]. It is important to highlight that this SNP is located near the internal ribosome entry site in the 5′UTR of the *bFGF* gene with an extremely important function. The substitution of C for U in the mRNA changes the structure, with important consequences in gene translation [46]. The analysis of mRNA expression of *bFGF* in a reduced number of our B-CLL samples show the same referenced association, where T/- patients have lower *bFGF* expression levels than CC carrier patients (Supplementary Figure S1). Additionally, higher bFGF concentration in plasma samples has been detected in IgVH mutated B-CLL patients (patients with better prognosis) in comparison with IgVH unmutated ones [12]. Data confirmed in our B-CLL patients series (Supplementary Figure S1).

The above suggests that high *bFGF* expression in B-CLL cells are related to good prognosis. Although this concept seems contradictory to generally held beliefs, the truth is that many manuscripts associate plasma high levels of VEGF factors, but not elevated bFGF factor, with worse prognosis. Wolowiec et al. found higher bFGF levels in patients with progressive disease than in stable ones, but this did not occur when the lymphocytosis doubling time (LDT) was compared, bFGF levels being lower in the LDT < 12 month versus the LDT > 12 month group indicating a relation between low bFGF level and fast progression [47]. Duensing et al. affirmed that enhanced bFGF plasma level was associated with adverse B-CLL: however they analyzed a low number of patients [48]. Other authors showed correlation between elevated intracellular levels of bFGF and the B-CLL state [49]. However, most recent studies have shown that only serum levels of VEGFs, but not bFGF, reflect clinic features of the tumor. The authors found no correlation between bFGF serum levels and disease progression [2,11].

## 5. Conclusions

Our results support the hypothesis that polymorphisms in the *VEGFA* and *bFGF* genes may have important implications in B-CLL. First, we observed that T allele carriers of rs3025039 *VEGFA* SNP had a significant protective effect for this disease, suggesting that this variant is a genetic marker of B-CLL protection, while its impact on the prognosis was unlikely. Secondly, our data suggest an association between the T/- rs1449683 *bFGF* variants and worse patient survival. To our knowledge, this is the first study to show an association between this SNP and survival. Changes in protein levels could be responsible for these observations; however, a broader case-control study of *bFGF* polymorphisms and predisposition to B-CLL, as well as an evaluation of *bFGF* expression levels in larger patient groups, are needed to determine the potential clinical impact of these findings.

## Figures and Tables

**Table 1:** Clinical and molecular characteristics of -cell chronic lymphocytic leukemia (B-CLL) patients.

B-CLL Patients (*n* = 204)		Number (%)
**Gender**	female	81 (39.7)
	male	123 (60.3)
Age ^a^, years	67 (40–93 ± 11)	
	<65	78 (38.2)
	>65	112 (54.9)
	n.d	14 (6.9)
Stage of the disease (RAI)	0	117 (57.3)
	I	48 (23.5)
	II	14 (6.9)
	III	2(1.0)
	IV	5 (2.5)
	n.d	18 (8.8)
Binet stage	A	163 (79.9)
	B	16 (7.8)
	C	7 (3.5)
	n.d	18 (8.8)
Adenopathy	Yes	65 (31.9)
	No	123 (60.3)
	n.d	16 (7.8)
Treatment	Yes	85 (41.7)
	No	74 (36.3)
	n.d	45 (22.0)
Transplant	Yes	15 (7.4)
	No	83 (40.7)
	n.d	106 (51.9)
Morphology	Typical	118 (57.8)
	Atypical	61 (29.9)
	n.d	25 (12.3)
Status	Dead	77 (37.7)
	Alive	40 (19.6)
	n.d	87 (42.7)
Cause of dead	Disease	34 (44.2)
	Other	43 (55.8)
Genetic lesions	Very low and low risk (del13q, NC, +12)	136 (66.7)
	Intermediated and high risk (del11q, del17p)	18 (8.8)
	n.d	50 (24.5)
LDH ^a^ level, UI/L		188 (100–1292 ± 158.032)
Hb level ^a^, g/dl		187 (5.2–132 ± 8.86)
Platelet count ^a^, ×10^9^/L		183 (21–382 ± 66.85)
Serumβ2microglobulin ^a^, g/L		181 (0.50–8.20 ± 1.28)
Peripheral blood lymphocytosis count ^a^, ×10^9^/L		186 (1.30–380.40 ± 45.09)
WBC count ^a^, ×10^9^/L		185 (4.30–42450 ± 52.16)
CD38 expression	Negative (<20%)	98 (48.0)
	Positive (≥20%)	74 (36.3)
	n.d	32 (15.7)
Zap-70 expression	Negative (<20%)	49 (24.0)
	Positive (≥20%)	80 (39.3)
	n.d	75 (36.7)
IgVH genes	M	43 (21.1)
	UM	7 (3.4)
	n.d	154 (75.5)

Abbreviations: ^a^ Mean at diagnosis (range and/or ± s.d); NC, normal karyotype; n.d, not determinated; WBC, white blood cell; M, mutated; UM, unmutated.

**Table 2:** Characteristics of the analyzed polymorphisms.

Gene	Chr	Chr Position	Ref SNP Number	Substitution	Minor Allele	Location	Taqman Assay
***VEGFA***	6	43860514	rs3025039	C/T	T	3′UTR	C_16198794_10
***VEGFA***	6	43837733	rs1109324	G/T	T	Promoter	C_8311589_10
***VEGFA***	6	43838622	rs1547651	A/T	T	Promoter	C_8311590_10
***VEGFA***	6	43831313	rs833052	A/C	A	Promoter	C_8311590_10
***VEGFR1***	13	-	-	C/T	T	Promoter	C_27837581_10
***bFGF***	4	123967536	rs1449683	C/T	T	Cds-syn	C_8837641_10

Chr, chromosome.

**Table 3:** Genotypic and allelic frequencies of *VEGF*, *VEGFR1* and *bFGF* polymorphisms and B-chronic lymphocytic leukemia risk.

Polymorphisms	Best Model ^1^	Genotype	Controls (*n* = 476)	Cases (*n* = 224)	OR (95% CI) ^a^	*p*-Value
**rs3025039** ***VEGFA***	A	C/C C/T T/T	355 (75.4%) 106 (22.5%) 10 (2.1%)	188 (83.9%) 34 (15.2%) 2 (0.9%)	0.61 (0.42–0.89)	**0.0075**
**rs1109324** ***VEGFA***	A	G/G G/T T/T	334 (70.9%) 127 (27.0%) 10 (2.1%)	162 (75.4%) 51 (23.7%) 2 (0.9%)	0.79 (0.56–1.10)	0.1600
**rs1547651** ***VEGFA***	A	A/A A/T T/T	325 (69.8%) 138 (29.6%) 3 (0.6%)	165 (73.7%) 54 (24.1%) 5 (2.2%)	1.35 (0.98–1.88)	0.0660
**rs833052** ***VEGFA***	O	C/C-A/A C/A	330 (72.1%) 128 (27.9%)	141 (77.9%) 40 (22.1%)	0.73 (0.49–1.10)	0.1300
**rs1449683** ***bFGF***	D	C/C C/T-T/T	374 (90.1%) 41 (9.9%)	175 (85.0%) 31 (15.0%)	1.62 (0.98–-2.66)	0.0630
**hcv 27837581** ***VEGFR1***		CC CT	451 (94.7%) 25 (5.3%)	217 (96.9%) 7 (3.1%)	0.57 (0.24–1.35)	0.1800

^a^ Odds ratio (OR) and 95% confidence interval (CI) for the single nucleotide polymorphism (SNP) main effect. ^1^ The Akaike information criterion (AIC) and Bayesian information criterion (BIC) were calculated to select the best inheritance model for each specific polymorphism. The preferred model is that with the lowest AIC and BIC value. Abbreviations: A, Log-additive; O, overdominant and D, dominant.

**Table 4:** Haplotype analysis of rs3025039, rs1109324, rs1547651 and rs833052 *VEGFA* SNPs and risk of developing B-CLL. The table shows haplotype frequency, odds ratio and *p*-value in genomic DNA from peripheral blood of all participants in this study. The haplotypes frequencies for the control group and the B-CLL group are shown in columns six and seven, respectively.

Haplotype Association with B-CLL Risk (*n* = 670)									
	rs3025039	rs1109324	rs1547651	rs833052	Group Control	Group B-CLL	Freq	OR (95% CI)	*p*-Value
1	C	G	A	C	0.5992	0.7333	0.6359	1.00	
2	T	G	A	C	0.113	0.0754	0.106	0.49 (0.31–0.79)	0.0036
3	C	T	T	C	0.1229	0.0295	0.0946	0.17 (0.08–0.36)	<0.0001
4	C	G	A	A	0.1106	0.0342	0.0895	0.21 (0.10–0.41)	<0.0001
5	C	T	T	A	0.0228	0.0615	0.0354	1.93 (0.92–4.02)	0.082

**Table 5:** Association of *VEGFA* and *bFGF* genotypes in B-CLL patients with significant risk factors.

Variant	Genotypes	Variable	OR (95% CI)	*p*-Value
rs3025039	C/T-T/T	Hb level (a)	3.91 (0.51–7.30)	**0.0200**
rs1449683	C/T-T/T	Genetic risk	4.46 (1.31–15.23)	**0.0250**
rs1547651	A/A	Hb level (a)	4.90 (2.24–7.57)	**4.00 × 10^−4^**
	A/T			
	T/T			
rs1547651	A/A	Adenopathy (a)	0.44 (0.21–0.92)	**0.0210**
	A/T			
	T/T			
rs1547651	A/A	Binet stage	0.13 (0.02–0.97)	**0.0059**
	A/T			
	T/T			
rs1547651	A/A	RAI stage	0.38 (0.18–0.79)	**0.0058**
	A/T			
	T/T			
rs1547651	A/A	Genetic risk	0.16 (0.02–1.22)	**0.0200**
	A/T			
	T/T			
rs1109324	G/G	Genetic risk	0.16 (0.02–1.21)	**0.0210**
	G/T			
	T/T			

Abbreviations: (a) Mean at diagnosis, OR: odds ratio; CI, confidence interval. Rai stages and Binet stages were categorized into early disease (0/I for Rai classification, A for Binet classification) and advanced disease (II/III/IV for Rai classification, B/C for Binet classification).

## Supplementary Materials

The following are available online at https://www.mdpi.com/2073-4425/11/6/686/s1, Figure S1: Expression of bFGF in B-CLL patients.
