# Supplementary material for: Clinical Relevance of +936 C>T VEGFA and c.233C>T bFGF Polymorphisms in Chronic Lymphocytic Leukemia
Source: Genes (Basel). 2020 Jun 23;11(6):686. doi: 10.3390/genes11060686 (PMC7349122; doi:10.3390/genes11060686)
Supplement: Supplementary file 1 [file genes-11-00686-s001.pdf]

Ballester et al.

Supplementary Figure 1.

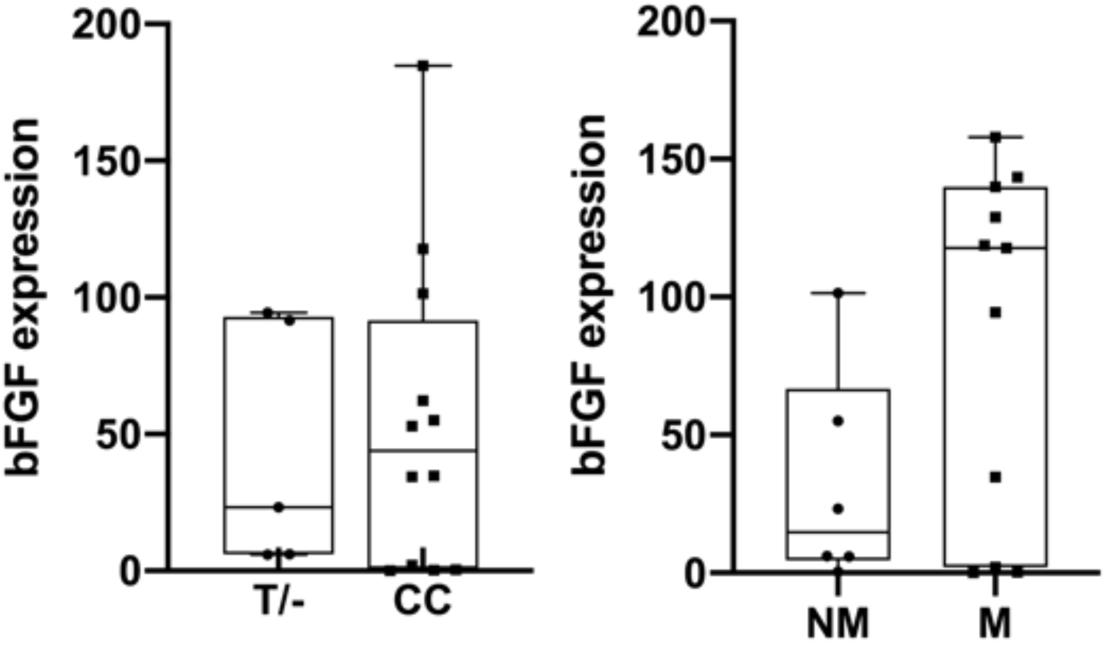

Supplementary Figure 1. **Expression of *bFGF* in B-CLL patients**. A) The *bFGF* expression was evaluated by Q-PCR in function of the rs1449683 bFGF genotype in 17 patients (T/- n=5, C/C n=12). B) The *bFGF* expression was evaluated in IgVH non-mutated patients (NM, n=6) and IgVH mutated patients (M, n=11).
